# Supplementary material for: Do Patients Residing in Provincial Areas Transport and Spend More on Cancer Treatment in Korea?
Source: Int J Environ Res Public Health. 2021 Sep 1;18(17):9247. doi: 10.3390/ijerph18179247 (PMC8431159; doi:10.3390/ijerph18179247)
Supplement: Supplementary file 1 [file ijerph-18-09247-s001.zip › ijerph-1317561-supplementary.pdf]

Supplementary Table S1. Mean days of care and healthcare costs between patients visiting hospitals located at an area different vs. identical to their area of residence

Variables	Area of residence vs. Location of hospital visited for treatment														
	Days of care							Costs							
	Different		P-value	Identical		P-value	P-value	Different		P-value	Identical		P-value	P-value
	Mean	SD		Mean	SD			Mean	SD		Mean	SD		
**Residing area**														
Capital area	127.1	173.2	<.0001	117.3	197.3	<.0001	0.0330	9.0	24.8	0.2708	8.8	26.6	0.0201	0.0205
Metropolitan cities	123.7	168.1		124.4	164.1			8.7	28.9		9.9	31.9		
Provincial areas	145.3	191.8		143.8	186.8			11.0	30.8		8.6	25.9		
**Institution of main treatment**														
General hospital	133.1	180.4	<.0001	135.2	200.7	<.0001	0.0137	10.8	31.3	<.0001	12.0	34.1	<.0001	0.2879
Hospital	146.4	221.1		142.5	167.4			6.7	10.8		6.9	13.2		
Long-term care hospital	273.3	188.2		263.4	186.8			14.8	10.5		14.6	14.7		
Clinic	94.6	139.5		81.9	130.6			1.6	3.9		1.1	2.6		
**Sex**														
Male	137.0	189.2	0.5146	130.4	202.9	0.5208	0.8660	10.5	29.1	0.8858	9.9	31.1	0.0139	0.2204
Female	134.6	172.6		126.6	162.0			9.3	28.3		7.7	22.5		
**Age**														
~69	122.3	154.9	0.0918	122.6	165.9	0.4933	0.5716	9.7	32.7	0.5340	9.0	28.3	0.1474	0.4434
70~74	123.3	163.0		116.7	170.0			8.6	23.9		8.1	24.6		
75~79	135.8	191.2		124.5	177.1			9.4	24.5		8.2	25.0		
80~	170.6	215.9		152.4	220.4			13.9	38.5		11.0	33.9		
**Type of insurance coverage**														
NHI self-employed	166.9	180.4	0.3785	163.0	200.3	<.0001	0.0406	12.5	29.1	0.4684	9.4	28.5	0.0395	0.7617
NHI employed	138.1	190.4		125.3	210.1			10.2	28.6		8.7	24.7		
Medical Aid	132.2	179.2		123.9	170.2			9.7	28.8		9.0	28.9		
**Economic status**														
Low	153.3	196.0	0.8224	141.3	199.2	0.7884	0.9560	11.4	31.8	0.8039	8.9	28.7	0.2253	0.5819
Mid-low	136.2	183.3		127.4	210.7			10.1	28.6		9.8	33.6		
Mid	132.9	167.2		125.0	168.2			9.9	30.3		9.3	26.2		
Mid-high	129.4	179.7		122.5	168.6			9.2	25.6		8.3	25.5		
High	127.3	181.0		120.2	169.9			9.3	27.0		8.6	23.3		
**Time since first diagnosis**														
~1 y	184.3	213.7	<.0001	189.1	230.8	<.0001	0.3785	17.5	40.9	<.0001	18.9	43.2	<.0001	0.9676
1y~2y	99.1	134.2		96.5	141.8			4.3	7.6		3.7	6.9		
2y~3y	96.2	140.6		92.5	140.3			3.7	6.7		3.2	7.3		
3y~4y	95.6	145.3		93.7	148.4			3.8	7.2		2.9	6.3		
4y~5y	91.8	151.2		90.9	145.4			3.7	8.8		2.8	6.7		
**Diagnosed cancer type**														
Gastric cancer	105.7	154.7	<.0001	106.8	15.9	<.0001	0.0002	6.3	19.2	<.0001	6.3	21.6	<.0001	0.0155
Colorectal cancer	119.0	159.5		111.9	185.8			7.9	31.0		6.9	26.9		
Hepatocellular carcinoma	181.1	222.9		172.0	201.3			17.7	37.0		16.0	38.9		
Lung cancer	193.2	219.0		175.2	215.5			16.0	32.3		14.1	31.0		

**Charlson Comorbidity Index (excluded Cancer)**														
~2	100.0	173.5	<.0001	97.2	180.5	<.0001	0.4427	9.0	24.8	<.0001	6.1	27.4	<.0001	0.3136
3~5	135.8	160.3		128.5	154.1			8.7	28.9		7.6	23.2		
6~	207.5	196.6		202.9	208.9			11.0	30.8		17.2	31.1		
**Died during observed year**														
Yes	379.2	261.4	<.0001	357.3	251.9	<.0001	0.0291	50.6	71.9	<.0001	46.7	62.9	<.0001	<.0001
No	109.9	150.2		102.2	156.8			5.7	12.9		4.6	14.7		

** NHI: National Health Insurance*

** Unit of cost: $1,000/ $1=1,131.5 KRW in 2015*

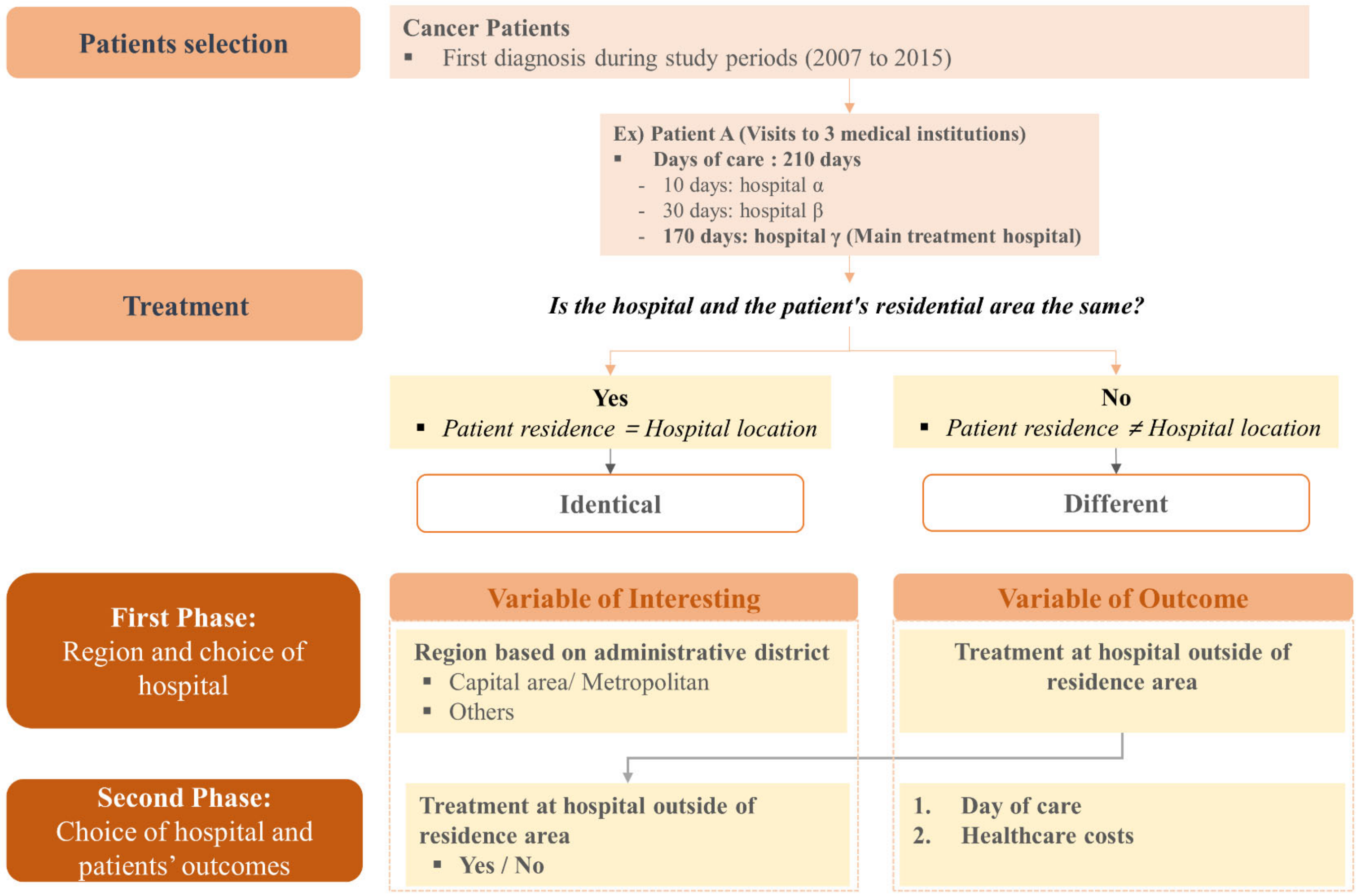

Supplementary Figure S1. Flow diagram of study methods
